# Risk Evaluation of Potentially Toxic Metals in Soils and Vegetables Surrounding Lanzhou City in Gansu Province, China

**DOI:** 10.3390/toxics13030158

**Published:** 2025-02-25

**Authors:** Hanru Ren, Jun Ren, Ling Tao, Xuechang Ren

**Affiliations:** 1 School of Environmental and Municipal Engineering, Lanzhou Jiaotong University, Lanzhou 730070, China; renjun@mail.lzjtu.cn (J.R.); taoling@mail.lzjtu.cn (L.T.); 2 Gansu Hanxing Environmental Protection Co., Ltd., Lanzhou 730070, China

**Keywords:** accumulation, vegetable, plantation base, bioconcentration, health

## Abstract

The potentially toxic metals in soil often cause secondary pollution of vegetables and pose a great threat to human health. Soil and vegetable samples were collected from eight different districts in the vegetable plantation base of Lanzhou city in Gansu province, and Zn, Cd, Cr, Cu, and Pb contents were determined using inductively coupled plasma atomic emission spectroscopy (ICP-AMS). The results suggested that the Cr and Zn contents of soils in the eight plantation bases were much higher than those of the other three metals. The metal contents showed significant differences among plantation bases and vegetable species, and the mean potentially toxic metal concentrations in soils exceeded background levels by 1.1~3.0 times. The accumulation of Cu in vegetables was significantly higher than that of other metals. Remarkable differences were found among the vegetables in the uptake abilities of Zn, Cd, Cr, and Cu. There were significant positive relationships between potentially toxic metal accumulation in vegetables and in soils. The results may be used to provide referential strategies and methods to minimize the impact of potentially toxic metals on human health through the consumption and cultivation of vegetables.

## 1. Introduction

The main sources of potentially toxic metals in the atmosphere, hydrosphere, soil and biota are industry, traffic, and polluted irrigation water, as well as several natural metals sources. Pollution caused by potentially toxic metals and their accumulation in the ecosystem poses serious problems worldwide [1,2]. Heavy metal contamination of water, soil, and crops is a severe environmental problem that has attracted worldwide attention. Agricultural soils are prone to contamination with potentially toxic metals from atmospheric deposition and various anthropogenic activities such as uncontrolled discharge from industrial and urban waste materials, sewage sludge, wastewater irrigation, and the excessive use of chemical fertilizers and pesticides [3,4]. Potentially toxic metals in soil pose a threat to the environment and food safety, and can have damaging effects on human and animal health [5,6,7,8,9,10,11]. The problem of heavy metal pollution in agricultural soil has been a longstanding hot topic of concern for many scientists and countries. Potentially toxic metals are persistent contaminants of the environment. They can change their chemical speciation, oxidation state, mobility, etc., and most potentially toxic metals do not undergo microbial or chemical degradation, which poses a significant threat to the environment and food safety. Potentially toxic metals can be harmful because of their potential to accumulate in different body parts of human beings and cause adverse health effects, even at low concentrations [12,13,14].

Vegetables are important edible crops and an essential part of the human diet. They are rich in nutrients required for human health and are an important source of carbohydrates, vitamins, minerals, and fiber. Potentially toxic metals in soils can be readily taken up by vegetable roots and can accumulate at high levels in the edible parts of vegetables, even at low levels in soil [15]. The uptake and bioaccumulation of potentially toxic metals in vegetables are influenced by a number of factors, such as climate, atmospheric deposition, the concentrations of potentially toxic metals in soil, the physicochemical properties of the soil in which the vegetables are grown, and the degree of maturity of the plants at the time of harvest [16,17,18,19]. Many phosphate rocks contain potentially toxic metals such as Pb and Cd, and high application rates of phosphorus fertilizer may not only increase soil P but also lead to the accumulation of metals above the maximum limit values [20,21].

There are many vegetable plantation bases on the outskirts of Lanzhou city, which is the principal vegetable production site for people living in Lanzhou and nearby. Some potentially toxic metals accumulate in soils and vegetables because of the use of chemical and organic fertilizers, pesticides, and growth-regulating agents. Potentially toxic metals in farmland soils from vegetable plantation bases surrounding Lanzhou city have been studied very little, and there has been no systematic investigation of potentially toxic metals in soil and vegetables in this area, nor of the accumulation and transfer characteristics of these potentially toxic metals. As potentially toxic metals in soil have a long residence time and are potentially dangerous, it is very important to study the accumulation of potentially toxic metals in soil and vegetables [22,23,24,25,26,27]. It is also vital to pay attention to food safety, as vegetables polluted by potentially toxic metals may bring about potential adverse effects on human beings [28,29].

In this study, field investigation and systematic sampling were carried out for Zn, Cd, Cr, Cu, and Pb in soils and vegetables in the vicinity of Lanzhou. The aims of this study were: (1) to measures the contents of five metals in soil and six main species of vegetables; (2) to assess the bioaccumulation factor of potentially toxic metals from soil by vegetables; (3) to investigate the relationship and difference between the metals in various species of vegetables; and (4) to assess the risk of potentially toxic metals in vegetables and soils in eight plantation bases surrounding Lanzhou City in Gansu Province, China.

## 2. Materials and Methods

### 2.1. Study Area

**Figure 1:**
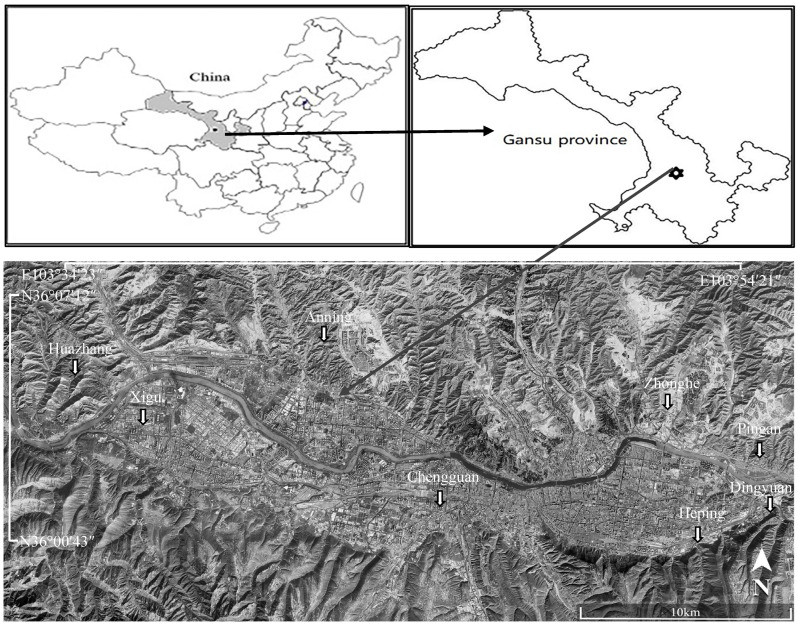
Location map of vegetable plantation bases (sampling sites) surrounding the Lanzhou city in Gansu province, China.

The study area comprised eight vegetable plantation bases located in the vicinity of Lanzhou city in Gansu province, China, at longitudes of 103°35′–103°55′ east and latitudes of 36°00′–36°08′ north. These bases are situated in the upper reaches of the Yellow River and are named Anning, Xigu, Chengguan, Heping, Dingyuan, Zhonghe, Pingan, and Huazhuang (Figure 1). The climate is subtropical monsoon, with an average annual temperature of 10.3 °C, average annual precipitation of 327.8 mm, and inadequate rainfall. Farmlands are irrigated with Yellow River water. The four distinct seasons and superior water heat conditions are conducive to crop growth.

### 2.2. Vegetables and Soils Sampling

Six vegetables and corresponding soil samples from each site were collected from April to October 2024. Six replicates from each sampling site were collected via systematic sampling. Soil samples were collected from the surface layer (0–20 cm), air-dried, and disaggregated. Identifiable stones and plant debris were removed and the samples were passed through a 2 mm mesh sieve. In order to determine heavy metal contents, a representative subsample of every soil sample was finely ground with porcelain mortar, passed through a 0.15 mm mesh sieve, and stored in closed plastic containers. The pH, EC (dS/m), organic matter (OM, %), and the content (mg/kg) of potentially toxic metals in the sampled farmland soils are presented in Table 1.

The most common vegetables from the soil sampling sites were selected for analysis. The six selected vegetables, locally grown and consumed by the population, included lettuce (*Lactuca sativa* L.), rape (*Brassica campestris* L.), leek (*Allium tuberosum* Rottl. ex Spreng), scallion (*Allium fistulosum* L.), cucumber (*Cucumis sativus* L.), and zucchini (*Cucurbita maxima* Duch. ex Lam.). The edible parts of the vegetables were picked, lightly washed with tap water to remove surface dust, then washed with deionized water, dried with filter paper, weighed, dehydrated in an oven at 105 °C for 2 h, and finally stored at 75 °C for 48 h.

### 2.3. Analysis of Samples

The soil pH was measured using a pH meter (PHS-3C) at a water-to-soil ratio of 1:5 (*w*/*v*), and the electrical conductivity (EC, dS/m) of soil samples was determined (conductivity meter DDSJ-308F) at a solid-water volume ratio of 1:5. The organic matter (OM) content of the soil was measured using the K_2_Cr_2_O_7_ volumetric method in an oil bath. The dried soil (sample size of 0.15 mm) was placed into a flask, 20 mL of aqua regia was added, and the flask was heated at 160 °C until the brown nitrogen oxides were exhausted. Then, soil samples were digested with an acid solution containing HCl, HNO_3_, and HClO_4_ on a hot plate, and heated at 190 °C until the soil was off-white. Finally, distilled water was added to the digested soil and filtered into a flask for the measurement of heavy metal concentration. The concentrations of Zn, Cd, Cr, Cu, and Pb in the digestion solution of soils were measured using an inductively coupled plasma mass spectrometer (ICP-AMS, Agilent 8900, Palo Alto, CA, USA) [6].

The dried plant samples were ground and placed into flasks, and 5 mL of nitric acid and 3 mL of hydrogen peroxide were added. Then, the flask was heated in an oven at 200 °C for approximately 6 h to obtain a transparent digestion solution. After cooling, the digestion solution was filtered and diluted with deionized water for the measurement of heavy metal concentration using an ICP-AMS [9].

### 2.4. Evaluation of Heavy Metal Pollution in Soil

The pollution levels of single and comprehensive heavy metals in soil were assessed using single-factor (P_i_) and Nemerow’s synthetic pollution index (P_n_). These indices are computed using Equations (1) and (2) [25,31]. Where P_i_ is the single-factor index, C_i_ is the concentration of each heavy metal, and S_i_ is the standard value for each heavy metal (Table 1). P_imax_ is the maximum of the single-factor index, and P_n_ is classified as either safe (P_n_ ≤ 0.7, Class I), precaution (0.7 < P_n_ ≤ 1.0, Class II), slightly polluted (1.0 < P_n_ ≤ 2.0, Class III), moderately polluted (2.0 < P_n_ ≤ 3.0, Class IV), or seriously polluted (P_n_ > 3.0, Class V).

(1) $$P_{i}=C_{i}/S_{i}$$

(2) $$P_{n}=\sqrt{(P_{imax}^{2}+{\bar{P}}^{2})/2}$$

The ecological risk index (RI), which comprehensively considers heavy metal content, environmental influence, and biotoxicity [31], was used to evaluate the risk of multiple heavy metals from soils to assess heavy metal pollution levels. The RI is computed by Equation (4), where $E_{r}^{i}$ represents the potential ecological risk index of each heavy metal, $C_{s}^{i}$ is the concentration of each heavy metal in soil, $C_{b}^{i}\;$ is the background value of heavy metal in soil in this region, and $T_{r}^{i}$ represents the toxic response coefficients. The values of Cu, Cr, Pb, Cd, and Zn were 5, 2, 5, 30, and 1, respectively. The RI classifications were low risk (RI < 150), moderate risk (150 ≤ RI < 300), considerable risk (300 ≤ RI < 600), and high risk (RI ≥ 600). The risk degrees of a single HM ($E_{r}^{i}$) were classified as low ecological risk ($E_{r}^{i}$ < 40), moderate ecological risk (40 ≤ $E_{r}^{i}$ < 80), considerable ecological risk (80 ≤ $E_{r}^{i}$ < 160), high ecological risk (160 ≤ $E_{r}^{i}$ < 320), and very high ecological risk ($E_{r}^{i}$ ≥ 320).

(3) $$E_{r}^{i}=T_{r}^{i}\times C_{s}^{i}/C_{b}^{i}$$

(4) $$RI=\sum_{i=1}^{n}E_{r}^{i}$$

The geo-accumulation index (I_geo_) is usually applied to assess the pollution level of heavy metals in soil and is calculated using Equation (5) [22], where K is introduced as a constant (1.5) to minimize the possible influence of background values due to geogenic variation. The pollution degrees of I_geo_ were classified as unpolluted (I_geo_ ≤ 0), slightly to moderately polluted (0 < I_geo_ ≤ 1), moderately polluted (1 < I_geo_ ≤ 2), moderately to heavily polluted (2 < I_geo_ ≤ 3), heavily polluted (3 < I_geo_ ≤ 4), heavily to extremely polluted (4 < I_geo_ ≤ 5), and extremely polluted (I_geo_ > 5).

(5) $$I_{geo}={log}_{2}(C_{s}^{i}/{(K\times C}_{b}^{i}))$$

### 2.5. Health Risk Assessment of Potentially Toxic Metals for Vegetable

The health risk of potentially toxic metals in vegetables can be assessed using the target hazard quotient (THQ) and hazard index (HI), proposed by the US Environmental Protection Agency (USEPA 2000). The THQ_ij_ was calculated using Equation (6) to assess the non-carcinogenic risks of vegetable j and heavy metal i, and HI_i_ was computed to estimate the potential risk of heavy metal i for all vegetables using Equation (7) [29]. C_ij_ is the concentration of heavy metal i in vegetable j (mg/kg, fresh weight); IR is the ratio of vegetable ingestion (kg/day) for lettuce (0.09), rape (0.08), leek (0.06), scallion (0.03), cucumber (0.12), and zucchini (0.09) [6]; EF is the exposure frequency (365 d/y); ED is the exposure duration (76.4 years, i.e., the life expectancy in China according to World Health Statistics 2018); BW represents the average body weight (70 kg); AT represents the average exposure time for non-carcinogenic effects (assuming 76.4 years, 365 days a year); and RfD is the daily reference dose of potentially toxic metals (mg/kg·d). The values of RfD for Cu, Cr, Cd, Zn and Pb were 0.04, 0.003, 0.001, 0.3, and 0.0035, respectively [19]. If the value of THQ or HI is ≤1.0, this indicates no obvious health risk to residents; on the contrary, non-carcinogenic risks are likely to occur when THQ or HI is > 1.0, and the health risk increases with an increase in THQ or HI.

(6) $${THQ}_{ij}=\frac{EF\times ED\times IR\times C_{ij}}{RfD\times BW\times AT}$$

(7) $${HI}_{i}=\sum_{j=1}^{m}{THQ}_{ij}$$

### 2.6. Bioconcentration Factors for Potentially Toxic Metals (BCF)

The bioconcentration factor (BCF) was used to evaluate the potential for heavy metal accumulation in vegetables related to heavy metal concentrations in soils. BCF was calculated using Equation (8) [29], where C_vegetable_ is the content of the heavy metals in the vegetable, and C_soil_ represents the heavy metal concentration in the soil.

(8) $$\mathrm{BCF}=\frac{C_{vegetable}}{C_{soil}}$$

### 2.7. Data Processing and Analyses

Statistical analysis was performed using STATISTICA 7.0 software. Summary statistics such as the mean and standard deviation were obtained in order to characterize soil properties and heavy metal contents. To assess the contamination status, the Government Standards for heavy metals in soils [30] and vegetables [32] were consulted as appropriate standard. Origin 2021 was used for graphical analysis, the two-way and one-way analysis of variance (ANOVA) to determine the effect of plantation base and species, and the calculation of the Pearson correlation coefficient. A multiple comparative analysis based on least significant difference (LSD) was used to separate statistically significant means with a mean variance of *p* < 0.05.

## 3. Results and Discussions

### 3.1. Levels of Potentially Toxic Metals in Soil

The accumulation of five potentially toxic metals in the soil and six vegetables from eight vegetable plantation bases surrounding Lanzhou city occurred at varying degrees. The ecological risk of potentially toxic metals in the Chengguan and Huazhuang regions was consistently lower than in other regions, whereas the Dingyuan and Xigu regions exhibited a significantly higher risk than other regions. These regions are the largest recipients of industrial wastewater, urban sewage, domestic waste, and solid waste soil in the city, and an important source of heavy metal pollution. The soils in the eight vegetable bases were all alkaline, as the soil pH values were higher than 8.0, with a mean of 8.35 and a range from 8.10 to 8.66. The EC of the soils in the eight planting bases was low, ranging from 0.26 to 0.57. The mean heavy metal concentrations (i.e., Zn, Cr, Pb, Cd, and Cu) in the soils of the eight different vegetable plantation bases exceeded the natural background levels. Specifically, the heavy metal levels were 1.1~3.0 times higher than the natural background levels. The concentrations of Zn, Cd, Cr, Cu, and Pb exceeded the natural background levels by 1.05, 3.00, 1.17, 1.18, and 1.32 times, respectively. The coefficient of variation (VC) indicates the degree of variation in the sampling sites, and the VC values for Zn, Cd, Cr, Cu, and Pb were 13.18%, 25.01%, 11.03%, 17.04%, and 9.04%, respectively. Mean heavy metal contents in soils followed the sequence: Cr > Zn ≫ Cu > Pb ≫ Cd. Cr and Zn contents were significantly higher than the other three metal contents. The Zn content was high in Chengguan district, at 78.66 mg/kg, and low in Anning district, at 54.10 mg/kg. The Cr content was high in Dingyuan district, with a value of 78.43 mg/kg, and low in Chengguan, at 55.03 mg/kg (Table 1).

Cr, Zn, Cu, Pb, and Cd levels in the soils of the eight sites were lower than the risk control standard for soil contamination of agricultural land [30], signifying that these potentially toxic metals in the soils were safe. Cd, Cr, and Pb contents were higher than soil background values. For Cd, the accumulation in lettuce in the Dingyuan site exceeded the grade II national standard of China. Relevant research has also confirmed that large and long-term application of organic fertilizers and phosphorus-containing fertilizers from livestock and poultry manure has led to serious accumulation of potentially toxic metals [33]. In addition, greenhouse vegetables are commonly cultivated with heavy metal-containing pesticides and films added during the planting process, which may also contribute to the accumulation of potentially toxic metals [34,35,36]. In this research, Cr, Pb, Cu, and Cd levels in the soil of the Anning region were greater than the background levels in soil. With the exception of the Anning, Heping, and Huazhuang sites, Zn levels were higher than the soil background value in the studied sites, and the Cu levels were higher than the soil background value in all sites except for the Huazhuang site (Table 1).

### 3.2. Pollution Assessment of Potentially Toxic Metals in Soil

**Figure 2:**
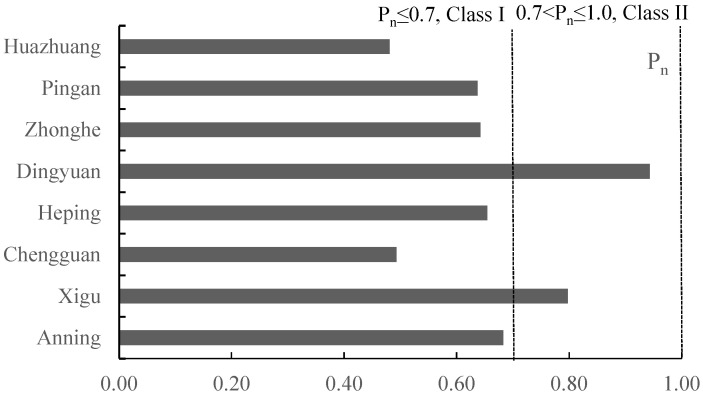
Pollution index (P_n_) for potentially toxic metals in farmland soils in eight vegetable plantation bases surrounding Lanzhou city in Gansu province, China.

The P_n_ indexes of the eight vegetable plantation bases showed lesser variations, and only the Xigu site exceeded the safety limit (Class I). Specifically, the P_n_ index of the Dingyuan site was higher than 0.7 and lower than 1 (Class II), showcasing slight pollution. The P_n_ values for all eight sites ranged from 0.48 to 0.94, spanning the range from precaution to slight pollution. The P_n_ of the eight vegetable plantation bases decreased in the following order: Dingyuan > Xigu > Anning > Heping > Zhonghe = Pingan > Chengguan > Huazhuang (Figure 2). The pollution levels of the soils indicated a certain degree of heavy metal pollution. Consequently, we assume that this might be due to unreasonable agricultural activities, such as the overuse of pesticides and chemical fertilizers, as well as the influence of the petrochemical industrial operations located near these vegetable bases [19].

The potential ecological risk indexes ($E_{r}^{i})$ of Zn, Cd, Cr, Cu, and Pb in the soils and the comprehensive potential ecological risk index (RI) of multiple potentially toxic metals were calculated, and the results indicated that Cd contributed to the majority of the ecological risk (Table 2). At the Chengguan and Huazhuang sites, the $E_{r}^{i}$ value for Cd was 62.50, suggesting a moderate ecological risk, and at the other six sites, the $E_{r}^{i}$ values for Cd were 80 ≤ $E_{r}^{i}$ < 160, posing a considerable ecological risk. The $E_{r}^{i}$ of Zn, Cr, Cu, and Pb at all of the studied bases were much less than 40, signifying a low ecological risk. For the five tested potentially toxic metals, the single contribution to the total potential ecological risk (RI) followed the order of Cd > Pb > Cu > Cr > Zn. Based on the ecological risk index for multiple potentially toxic metals, the RI of the total samples exhibited low risk (Table 2).

**Figure 3:**
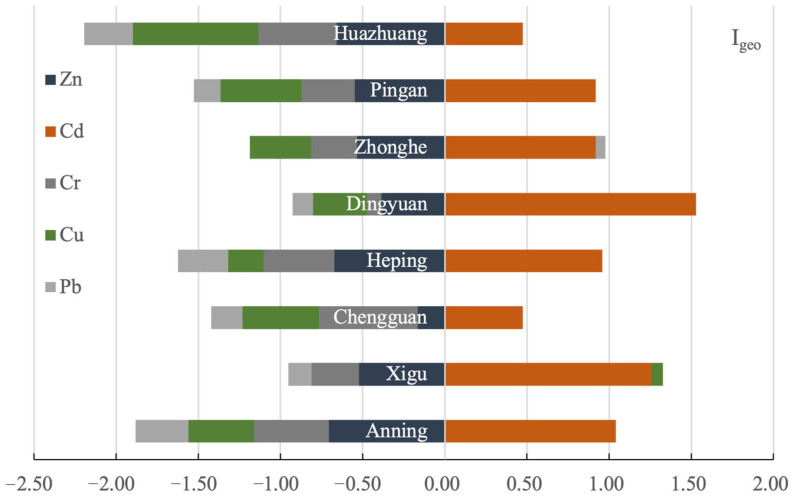
The geo-accumulation index (I_geo_) for potentially toxic metals from farmland soils in eight vegetable plantation bases surrounding Lanzhou city in Gansu province, China.

The negative I_geo_ values of Zn and Cr indicated that the eight vegetable plantation bases were free from Zn and Cr contamination. The I_geo_-Cu in all vegetable bases, except the Xigu site, and I_geo_-Pb in all vegetable bases, except the Zhonghe site, were less than zero. Additionally, the I_geo_-Cu in the Xigu site and I_geo_-Pb in the Zhonghe site were shown to be within the range of 0 < I_geo_ ≤ 1, suggesting that the degree of pollution with Cu and Pb in these two sites were representative of slight to moderate pollution. The I_geo_-Cd showed a larger spatial variation; the I_geo_-Cd values at the Chengguan, Heping, Zhonghe, Pingan, and Huazhuang sites were 0 < I_geo_ ≤ 1, indicating that the degree of pollution with Cd at these five sites was slight to moderate. The I_geo_-Cd values at the Anning, Xigu, and Dingyuan sites were in the range of 1 < I_geo_ ≤ 2, indicating that these sites were moderately polluted with Cd (Figure 3).

### 3.3. Levels of Potentially Toxic Metals in Vegetables

**Figure 4:**
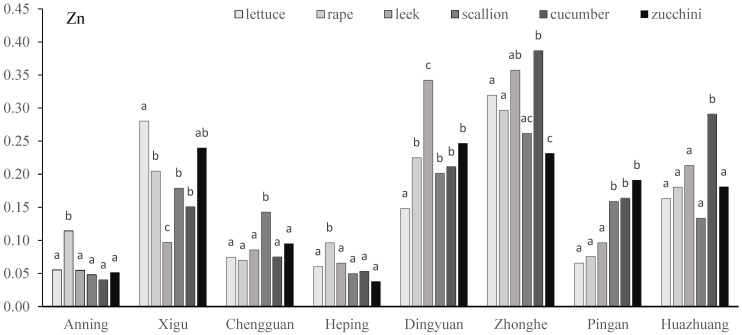
Zn concentration (mg/kg) of vegetables in eight vegetable plantation bases surrounding Lanzhou city in Gansu province, China. Lowercase letters above bars indicate a difference between different vegetables; the same letter indicates no significant difference.

A two-way ANOVA showed that the contents of Zn, Cd, Cr, and Cu were significantly affected by sites and vegetable species, and the interactions between sites and vegetables were not significant (Table 3). The Zn content of vegetables varied significantly within species in each vegetable base. At the Anning and Heping sites, the Zn content in lettuce was significantly higher than in other vegetables. At the Xigu site, the Zn contents in rape, scallion, and cucumber showed no significant difference, and they were significantly higher than in leek, and lower than in lettuce. At the Chengguan site, the Zn content in scallion was significantly higher than in other vegetables, and there was no significant difference between the other five vegetables. At the Dingyuan site, no significant difference in Zn content was observed among rape, scallion, cucumber, and zucchini, and the Zn content in these four vegetables was significantly higher than lettuce and lower than leek. At the Zhonghe site, the highest Zn content appeared in cucumber, and zucchini showed the lowest accumulation capacity. At the Pingan site, the Zn contents of lettuce, rapeseed, and leek did not show any significant difference, and they were significantly higher than those of the other three vegetables, with no significant differences observed between scallion, cucumber, and zucchini. At the Huazhuang site, the Zn content in cucumber was significantly higher than in other vegetables, and there was no significant difference among other five vegetables (Figure 4).

**Figure 5:**
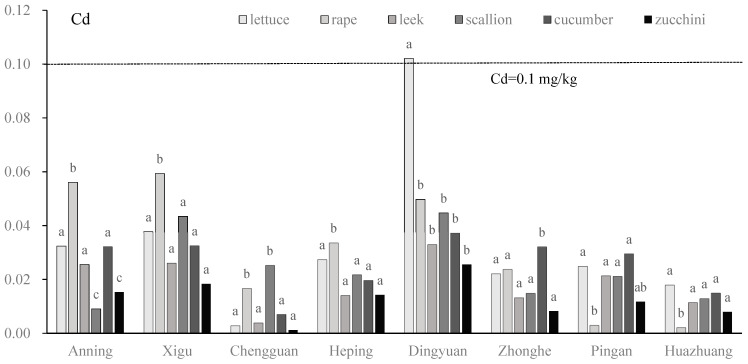
Cd concentration (mg/kg) of vegetables in eight vegetable plantation bases surrounding Lanzhou city in Gansu province, China. Lowercase letters above bars indicate the difference between different vegetables; the same letter indicates no significant difference [32].

Cd contents of vegetables were significantly different among vegetables across the eight vegetable plantation bases. At the Xigu and Heping sites, the Cd contents in rape were significantly higher than in other vegetables, and there was no significant difference among the other five vegetables; at the Pingan and Huazhuang sites, entirely opposite results were observed. At the Anning site, the Cd contents of lettuce, leek, and cucumber were not significantly different; they were significantly higher than scallion and zucchini, and lower than rape. At the Chengguan site, lettuce, leek, cucumber, and zucchini consistently showed lower Cd contents, and the Cd contents of rape and scallion were significantly higher than the other four vegetables. At the Dingyuan site only, the Cd content of lettuce exceeded the Chinese government standards for potentially toxic metals in vegetables [32], and it was significantly higher than the other five vegetables. There were significant differences among the remaining five vegetables. At the Zhonghe site, the Cd contents of lettuce, rape, leek, scallion, and zucchini exhibited no significant difference, and they were significantly lower than cucumber (Figure 5).

**Figure 6:**
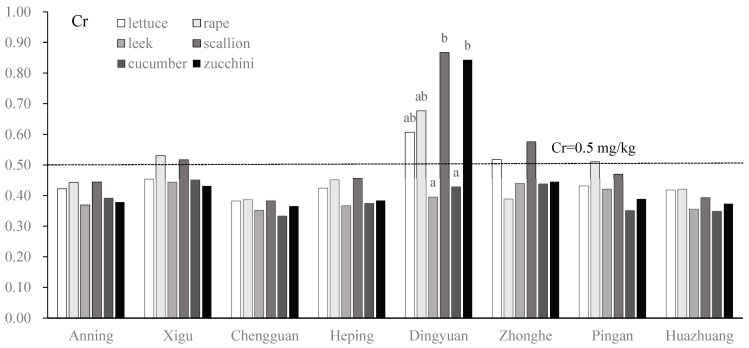
Cr concentration (mg/kg) of vegetables in eight vegetable plantation bases surrounding Lanzhou city in Gansu province, China. Lowercase letters above bars indicate the difference between different vegetables; the same letter indicates no significant difference [32].

**Figure 7:**
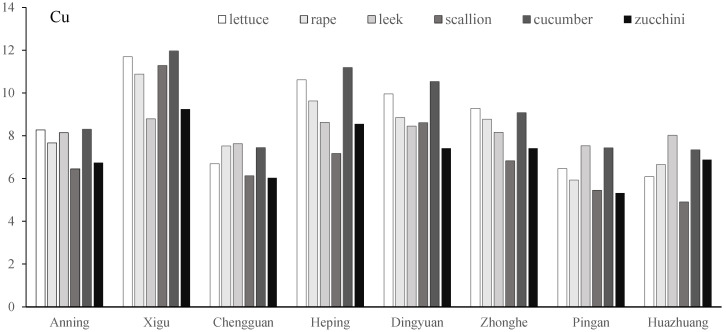
Cu concentration (mg/kg) of vegetables in eight vegetable plantation bases surrounding Lanzhou city in Gansu province, China.

There were significant differences in the Cr contents of the six vegetables at the Dingyuan site (One-way ANOVA: F_5,25_ = 4.59, *p* < 0.01), and no significant difference manifested among the other seven sites. The Cr contents of rape and scallion at the Xigu site, lettuce and scallion at the Zhonghe site, and rape at the Pingan site slightly exceeded the National Food Safety Standards for contaminant limits in China [32]. More serious Cr pollution was found at the Dingyuan site; the Cr contents of lettuce, rape, scallion, and zucchini exceeded the standard, while leek and cucumber were relatively safe (Figure 6). The Cu contents of the six vegetables across the eight vegetable plantation bases did not show a significant difference; lettuce and cucumber accumulated the most Cu across most sites, and scallion showed lower Cu uptake (Figure 7).

**Figure 8:**
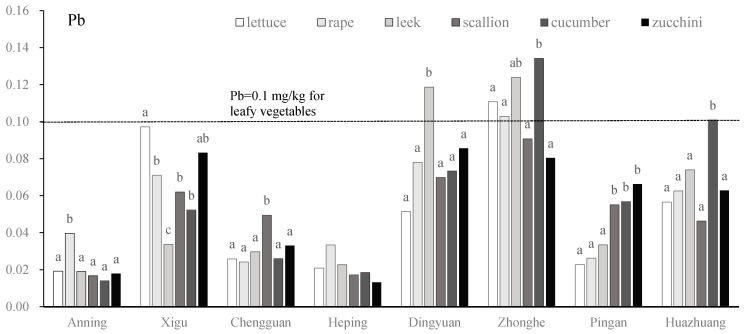
Pb concentration (mg/kg) of vegetables in eight vegetable plantation bases surrounding Lanzhou city in Gansu province, China. Lowercase letters above bars indicate the difference between different vegetables; the same letter indicates no significant difference [32].

There were significant differences in the Pb contents among vegetables in seven of the bases. At the Anning site, the Pb content in rape was significantly higher than in the other vegetables, and there were no significant differences among the other five vegetables. At the Xigu site, the Pb contents of rape, scallion, and cucumber were significantly higher than leek and lower than lettuce. At the Chengguan site, scallion exhibited the highest Pb content to a significant degree; no significant differences were found among the other five vegetables. There was a significant difference in Pb contents among the six vegetables at the Heping site. At the Dingyuan site, the Pb content of leek exceeded the Chinese government standards for heavy metals in vegetables [32], and was significantly higher than the other five vegetables, between which there were significant differences. At the Zhonghe site, the Pb contents of scallion, cucumber, and zucchini showed no significant differences, and were significantly higher than lettuce, rape, and leek. At the Huazhuang and Pingan sites, the Pb content of leek was significantly higher than in the other five vegetables, and slightly exceeded government standards (Figure 8).

The detected sequence of Cr, Zn, Cu, Pb, and Cd was significantly affected by sites and species. From the linear model regression analysis, remarkable significant positive relationships were discovered between metal accumulation in vegetables and in soils for Zn in leek, cucumber, and zucchini, Cd in lettuce, scallion, and cucumber, Cr in leek and zucchini, Cu in all vegetables except zucchini, and Pb in lettuce and scallion. The heavy metal contents in the vegetables were relatively low, and only one leafy vegetable sample had Cd levels exceeding the national standard, indicating that the accumulation of potentially toxic metals in vegetables was influenced by conditions other than the total amount of potentially toxic metals in the soil. There are many factors that affect the uptake of potentially toxic metals in vegetables, such as soil physicochemical characteristics, the speciation distribution of heavy metals in soil, biological effectiveness, vegetable varieties, planting management conditions, and spatial differences [31,33,34]. Therefore, when investigating heavy metal accumulation in vegetables, more attention should be paid to the possible effects of factors other than the heavy metals in the soil.

### 3.4. The Health Risks of Potentially Toxic Metals in Vegetables

**Figure 9:**
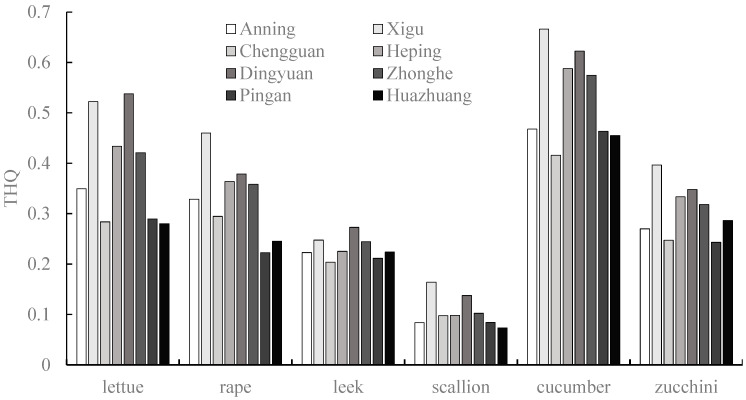
Target hazard quotients (THQs) of different vegetables in eight vegetable plantation bases surrounding Lanzhou city in Gansu province, China.

**Figure 10:**
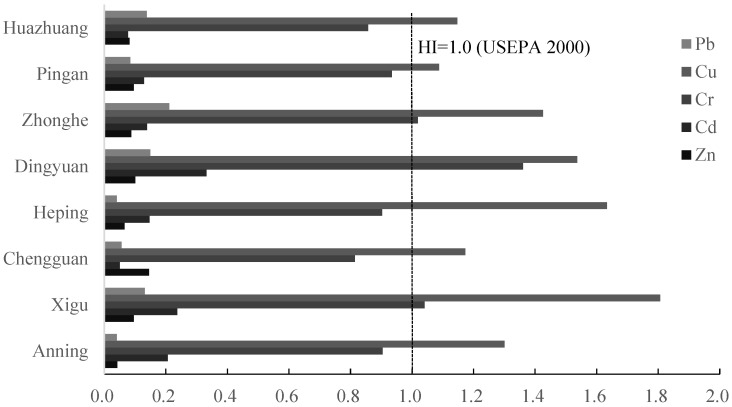
Hazard index (HI) of vegetables in eight vegetable plantation bases surrounding Lanzhou city in Gansu province, China.

The target hazard quotient (THQ) and hazard index (HI) were applied to assess the human health risk of potentially toxic metals from vegetable growth in the vegetable bases surrounding Lanzhou city in Gansu province, China. There were some differences in the THQs because the potentially toxic metals that contaminated the soils were different across the eight vegetable plantation bases. The THQs of potentially toxic metals for each vegetable from the Xigu, Dingyuan, Heping, and Zhonghe sites exhibited consistently higher values. All of the THQ values of potentially toxic metals for the eight vegetable bases were less than 1, indicating no obvious health risk to the surrounding residents consuming potentially toxic metals via an individual vegetable (Figure 9). The HI values of Cu across all of the vegetable bases were higher than 1, suggesting that all of the sites were likely to incur some degree of health risk related to Cu. The HI values also exceeded 1 for Cr at the Zhonghe, Dingyuan, and Xigu sites, indicating a degree of Cr-related health risk at these three sites (Figure 10).

Hazard index (HI) has been approved as an important index of health risk assessment. It is used to assess the health risk associated with uptake of heavy metal in food crops. In this study area, it was as follows: Cu > Cr > Cd > Zn > Pb. The HIs of all of the potentially toxic metals investigated in this study were < 1, except for Cu across all sites and Cr at the Zhonghe, Dingyuan, and Xigu sites. In the present study, the THQ indexes of not only the non-essential but also the essential metals were investigated. Different vegetable species exhibit different accumulation capacities for potentially toxic metals. It has been reported that Cd uptake in leafy vegetables is greater than in non-leafy vegetables. In this research, significant differences were not found in the contents of potentially toxic metals in the edible parts of different vegetable types. Cucumber had higher concentrations and THQs of potentially toxic metals, and may be classed as a “high accumulator” of Cd. Lower THQs of potentially toxic metals were found in scallion, classed as a “low accumulator” [9]. This suggests that low accumulators are suitable for planting in heavy metal-polluted soil, while high accumulators are unsuitable. The strong accumulation ability of leafy vegetables for potentially toxic metals and the resulting high concentrations within the vegetables could possibly be due to the leaves being the main parts of the vegetables used for photosynthesis, and more metals flowing to the leaves by strong transpiration. However, fruits and vegetables accumulate more potentially toxic metals in the edible part, which may be due to the longer growth period and thus longer accumulating time for potentially toxic metals [17]. Furthermore, atmospheric deposition might be one of the reasons for metal uptake of leafy vegetables through leaf stomata [24].

Both Cu and Zn are important nutrient elements for humans, and are considered to pose much lower health risks than Pb, Cd, and Cr [11,16,26]. Poor health can be caused by a lack of these required metal elements, but excessive ingestion can also pose health risks. Presently, there are several methods to estimate the potential health risks of potentially toxic metals, covering both carcinogenic and non-carcinogenic effects. Non-cancer risk assessment is typically based on the THQ method, which is a ratio of the determined dose of a pollutant to the reference oral dose [37]. THQ values are associated with intake of potentially toxic metals, exposure period, body weight, and reference oral doses. Vegetables comprise only a part of human diets, and in addition to vegetable consumption, consumption of rice, meat, fish, and tobacco can also lead to the intake of large amounts of potentially toxic metals [38,39].

### 3.5. The Bioconcentration Factor of Potentially Toxic Metals

The BCF of each vegetable showed significant differences among five of the assessed potentially toxic metals, and among six vegetables for Zn and Cd. All six vegetables exhibited a consistent uptake capacity for Cr, Cu, and Pb. The BCFs of Zn in lettuce, rape, and cucumber showed no significant difference, and were significantly higher than in the other three vegetables. Leek, scallion, and zucchini also showed no significant difference for Zn. The BCFs of scallion and cucumber for Cd were significantly higher than leek and zucchini, and lower than lettuce and rape. The BCFs of different potentially toxic metals exhibited various responses across the various examined vegetables. The BCFs of Cu in all vegetables was much higher than other four potentially toxic metals, and lettuce did not show a significant difference for Zn, Cd, Cr, and Pb. The BCFs of rape, leek, scallion, cucumber, and zucchini for Zn and Cd were higher than for Cr and Pb, and there were significant differences between Zn and Cd, and between Cr and Pb (Table 4).

### 3.6. Correlation Analysis

**Figure 11:**
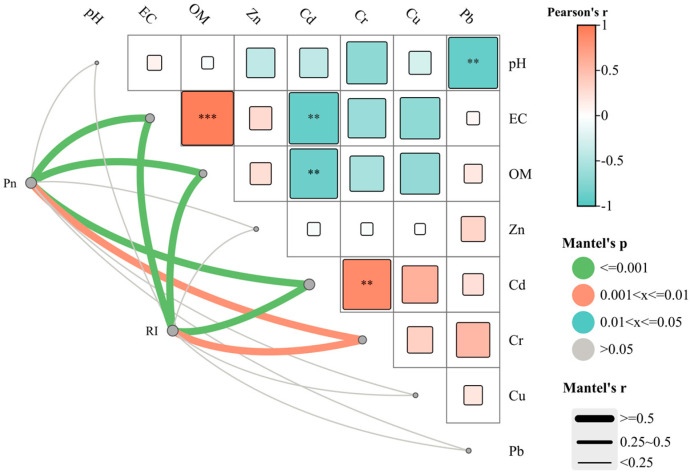
Correlation plot of P_n_, RI, physicochemical properties and potentially toxic metals in soils across eight vegetable plantation bases surrounding Lanzhou city in Gansu province, China. Correlation: ** at 0.01 level, *** at 0.001 level.

**Figure 12:**
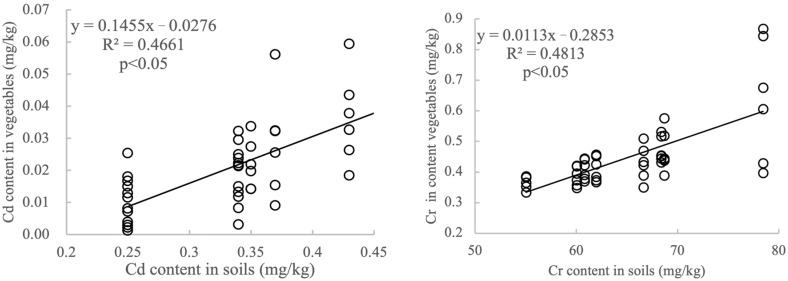

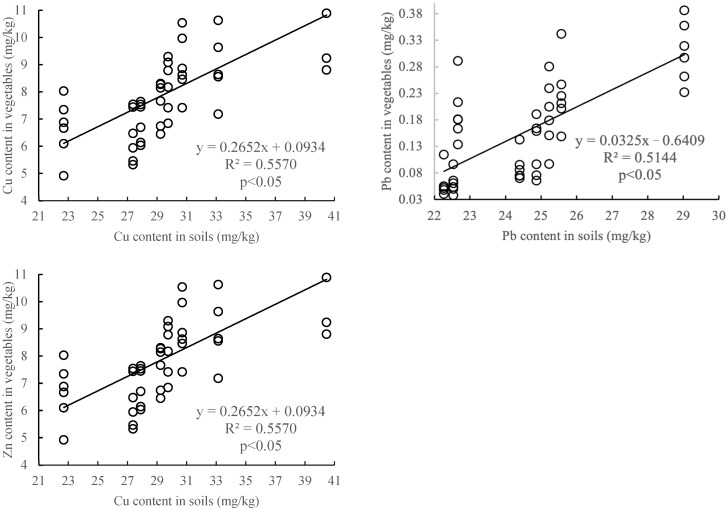
Correlation between the contents of potentially toxic metals in the vegetables with that in the soils across eight vegetable plantation bases surrounding Lanzhou city in Gansu province, China.

The physicochemical parameters (EC and OM) of soils from the eight vegetable bases showed a strong positive correlation (r = 0.96) at *p* < 0.001. There were extremely significant correlations at *p* < 0.01 between pH and Pb (r = −0.90), and between EC and Cd (r = −0.84). A significant correlation at *p* < 0.05 was found between OM and Cd (r = −0.78). RI and P_n_ both exhibited a significant correlation with EC, OM, and levels of Cr and Cd (Figure 11). The significant correlations between the heavy metal contents in soil and in the edible parts of vegetables were observed according to the correlation analysis (*p* < 0.05) for five potentially toxic metals and eight vegetables (Figure 12). It was speculated that the potentially toxic metals in vegetables may come from the soil, which may indicate that potentially toxic metals in the vegetables tend to accumulate in the edible parts, but not in the roots and other vegetable parts [17,31,40].

## 4. Conclusions

Cr and Zn levels in soils across the eight studied vegetable bases were significantly higher than Cd, Pb, and Cu. There were different levels of potentially toxic metals pollution discovered, and the soils at the Dingyuan and Xigu sites exhibited higher levels of potentially toxic metals pollution and were moderately polluted by Cd. The ecological risk level followed the order of Cd > Pb > Cu > Cr > Zn for all vegetable bases. Some measures need to be taken to control the ecological risks of Cu and Cd in the soil of plantation bases surrounding Lanzhou city.

Cu and Zn in the six vegetables species from all eight plantation bases did not exceed the Chinese government standards from for potentially toxic metals in vegetables [32]. At the Xigu, Dingyuan, and Zhonghe sites, the contents of Cd, Pb, and Cu in vegetables exceeded this standard, representative of certain food safety risks. Most vegetables from the Xigu, Dingyuan, Heping, and Zhonghe sites did not present obvious health risks. The levels of Cu and Cr observed at the Zhonghe, Dingyuan, and Xigu sites were suspected as posing a health risk due to excessive hazard index values. The vegetables examined exhibited different accumulation abilities for different potentially toxic metals, and Cu and Cd were more easily accumulated in edible parts by vegetables. Higher EC and OM content in soils appeared to promote the uptake of potentially toxic metals, and there were significant correlations between the content of potentially toxic metals in the soils and in the vegetables grown in them [31].

The main potentially toxic metal sources in vegetable plantation bases are human activities and natural sources. Referential strategies and methods should be adopted to minimize the impacts of potentially toxic metals to human health caused via consumption and cultivation of vegetables in the vegetable bases surrounding Lanzhou city. The calculation of the risk assessment model was based on the sampling data, which could lead to uncertainties due to the lack of comprehensive data surrounding soil and local vegetables in this research. There may be uncertainties caused by factors related to regional vegetable planting methods and land use modes, which would inevitably increase the uncertainty of the results. Parameters published in this research may also need to be updated to more accurately represent current conditions [41]. Although the abovementioned factors may introduce some uncertainties, this investigation can still provide valuable information for better control of environmental risks and the adjustment of vegetable planting modes by local governments and farmers. The results can provide a scientific basis for reducing harm in cultivation, and a more scientific method for the risk assessment of heavy metal contamination in vegetables by using a comprehensive risk index to jointly evaluate the impact of potentially toxic metals in soil and vegetables on human health.

## Figures and Tables

**Table 1:** The pH, EC (dS/m), organic matter (OM, %) and the content (mg/kg) of potentially toxic metals in farmland soils in eight planting bases surrounding Lanzhou city in Gansu province, China (*n* = 6).

Site	pH	EC	OM	Zn	Cd	Cr	Cu	Pb
Anning	8.7 ± 1.2	0.31 ± 0.06	1.43 ± 0.08	54.10 ± 4.32	0.37 ± 0.07	60.80 ± 6.58	29.24 ± 3.22	22.27 ± 1.65
Xigu	8.2 ± 0.8	0.27 ± 0.04	1.38 ± 0.06	61.37 ± 5.39	0.43 ± 0.08	68.40 ± 8.35	40.46 ± 5.29	25.23 ± 3.26
Chengguan	8.4 ± 1.5	0.57 ± 0.09	1.95 ± 0.21	78.66 ± 6.84	0.25 ± 0.06	55.03 ± 6.39	27.90 ± 3.27	24.40 ± 1.55
Heping	8.6 ± 0.6	0.37 ± 0.03	1.42 ± 0.10	55.31 ± 4.37	0.35 ± 0.03	61.98 ± 5.29	33.14 ± 2.33	22.53 ± 1.68
Dingyuan	8.2 ± 1.3	0.26 ± 0.02	1.28 ± 0.09	67.48 ± 7.85	0.52 ± 0.04	78.43 ± 9.64	30.71 ± 2.45	25.57 ± 1.79
Zhonghe	8.1 ± 0.7	0.49 ± 0.06	1.87 ± 0.08	60.88 ± 8.24	0.34 ± 0.02	68.73 ± 8.78	29.75 ± 2.36	29.03 ± 2.04
Pingan	8.3 ± 0.5	0.41 ± 0.05	1.76 ± 0.15	60.28 ± 6.55	0.34 ± 0.02	66.68 ± 6.25	27.38 ± 3.15	24.87 ± 2.16
Huazhuang	8.5 ± 0.4	0.56 ± 0.06	1.96 ± 0.12	55.86 ± 4.35	0.25 ± 0.01	60.02 ± 5.34	22.69 ± 1.98	22.66 ± 1.22
Mean	8.36	0.41	1.63	61.74	0.36	65.01	30.16	24.57
Background value of heavy metal in soil ^a^				58.74	0.12	55.66	25.68	18.56
Risk standard of heavy metal for soil (S_i_) ^b^				300.00	0.60	250.00	100.00	170.00

^a^ Soil background values in Gansu province (soil background values of Chinese elements, 1990); ^b^ Soil environment quality [30].

**Table 2:** Ecological risk index ($E_{r}^{i})$ for single heavy metals and RI for multiple potentially toxic metals from farmland soils in eight vegetable plantation bases surrounding Lanzhou city in Gansu province, China.

Site	$E_{r}^{i}$					RI
	Zn	Cd	Cr	Cu	Pb	
Anning	0.92 ± 0.01	92.47 ± 9.65	2.18 ± 0.12	5.69 ± 0.46	6.00 ± 0.51	107.30
Xigu	1.04 ± 0.02	107.48 ± 13.45	2.46 ± 0.16	7.88 ± 0.51	6.80 ± 0.52	125.68
Chengguan	1.34 ± 0.03	62.46 ± 6.35	1.98 ± 0.15	5.43 ± 0.42	6.57 ± 0.60	77.82
Heping	0.94 ± 0.01	87.49 ± 5.26	2.23 ± 0.24	6.45 ± 0.41	6.07 ± 0.56	103.19
Dingyuan	1.15 ± 0.02	130.16 ± 12.24	2.82 ± 0.10	5.98 ± 0.48	6.89 ± 0.58	146.83
Zhonghe	1.04 ± 0.02	85.22 ± 8.25	2.47 ± 0.14	5.79 ± 0.50	7.82 ± 0.62	102.12
Pingan	1.03 ± 0.01	85.15 ± 7.29	2.40 ± 0.07	5.33 ± 0.56	6.70 ± 0.71	100.45
Huazhuang	0.95 ± 0.01	62.48 ± 7.11	2.16 ± 0.11	4.42 ± 0.61	6.10 ± 0.66	76.13

**Table 3:** Analysis of variance for the effects of different sites, vegetables, and their interaction on heavy metal accumulation in vegetables.

Metal	Source of Variation	df	*F*-Value	*p*
Zn	site	7	12.98	<0.001
	vegetable	5	7.98	<0.001
	site × vegetable	35	0.76	0.8346
Cd	site	7	5.27	<0.001
	vegetable	5	4.66	<0.001
	site × vegetable	35	1.42	0.0663
Cr	site	7	12.12	<0.001
	vegetable	5	5.20	<0.001
	site × vegetable	35	1.40	0.0748
Cu	site	7	17.25	<0.001
	vegetable	5	6.72	<0.001
	site × vegetable	35	0.78	0.8073
Pb	site	7	30.29	<0.001
	vegetable	5	0.50	0.7751
	site × vegetable	35	1.70	<0.05

**Table 4:** Bioconcentration factors (BCFs) of potentially toxic metals for vegetables from eight vegetable plantation bases surrounding Lanzhou city in Gansu province, China. Values with the same lowercase letters indicate no significant difference among vegetables for each potentially toxic metal, and values with capital letters indicate no significant difference among potentially toxic metals for each vegetable at 5% level of probability by Duncan’s multiple comparison test. ***, ** indicate very significant difference (*p* < 0.001, *p* < 0.01).

Vegetable	Zn	Cd	Cr	Cu	Pb	*F*-Value
lettuce	0.0755 Aa	0.0840 Aa	0.0070 A	0.2844 B	0.0058 A	79.8301 ***
rape	0.0726 Aa	0.1081 Aa	0.0073 B	0.2733 C	0.0063 B	41.2892 ***
leek	0.0478 Ab	0.0496 Ac	0.0061 B	0.2763 C	0.0065 B	249.7786 ***
scallion	0.0442 Ab	0.0665 Ab	0.0078 B	0.2329 C	0.0058 B	141.0772 ***
cucumber	0.0715 Aa	0.0699 Ab	0.0060 B	0.3035 C	0.0068 B	335.2488 ***
zucchini	0.0494 Ab	0.0336 Ac	0.0068 B	0.2402 C	0.0063 B	273.8285 ***
*F*-value	5.1880 **	13.3202 ***	2.0736	1.7582	0.0952	

## Data Availability

All relevant data are within the paper.
